# Pregnancy in dialysis patients: a case series

**DOI:** 10.1186/1752-1947-2-10

**Published:** 2008-01-20

**Authors:** Khalid A Al-Saran, Alaa A Sabry

**Affiliations:** 1 Prince Salman Center for Kidney Diseases, Riyadh, Kingdom of Saudi Arabia

## Abstract

Fertility is markedly reduced in patients with chronic renal failure. For women with pre-existing renal disease, pregnancy is associated with an increased rate of fetal complications and a considerable risk of renal disease progression. Due to substantial improvements in antenatal and neonatal care, fetal outcome has improved considerably in the last two decade.

A Saudi survey which examined the frequency of pregnancy among women in end stage renal disease (ESRD) and undergoing regular hemodialysis (HD), showed an incidence of 7% over a five year period (1.4 per year). This may reflect the cultural endorsement of having offspring.

We hereby report 2 cases of successful pregnancy managed at the Prince Salman Center for Kidney Diseases (PSCKD).

## Case presentation

### Case 1

A 37 year old Saudi female, 8^th ^gravida with a history of a single 2^nd ^trimester abortion and six living offsprings. The patient was started on regular HD at PSCKD in January 2006. Three months after maintenance hemodialysis she presented with abdominal distension and 4 weeks history of amenorrhea. She was found to be pregnant after measurement of serum HCG, confirmed by a pelvi-abdominal ultrasonography. Her dialysis prescription consisted of 3 extended weekly sessions (7 hours each) due to her refusal of daily dialysis. Her eKt/V was between 3 to 3.7 (she was passing 400 ml urine/day with estimated GFR of 4.9 ml/min/1.73 m^2^). Her normalized protein catabolic rate (nPCR) was 1.46; blood gas analysis revealed a pH of 7.36, HCO3 of 24.2 meq/L, serum calcium 2.26 mmol/L and serum phosphate 1.17 mmol/L. As part of her medication the required dose of erythropoietin was increased from a mean weekly dose of 8000 units to 14000 units during pregnancy to maintain a hemoglobin level of 10 gm/dl. Iron was also increased from oral ferrous fumarate (100 mg/day) to IV iron saccharate (100 mg/weekly). She also received Calcium carbonate 1500 mg/day as a phosphate binder, multivitamins and folic acid. Her mean pre-dialysis blood urea nitrogen (BUN) was 15.13 mg/dl. The blood pressure was controlled without antihypertensive medication. A boy weighing 2.3 kg was delivered by vaginal delivery in the 30th week of gestation with uneventful neonatal period.

### Case 2

A 36 year old female patient, 9^th ^gravida with 4 living offsprings and four abortions. Her first pregnancy was complicated by pre-eclampsia, nevertheless it was completed successfully and the following 3 successful pregnancies were uncomplicated. She had a history suggestive of chronic glomerulonephritis.

During the first trimester of her 9^th ^pregnancy (March 2006), she developed lower limb oedema and felt unwell with persistent nausea and vomiting. Her urinalysis revealed proteinuria 3+ and her biochemical investigations revealed high serum creatinine (15.56 mg/dl) and a BUN level of 74 mg/dl. The patient was diagnosed as a case of ESRD. Renal biopsy was not done in view of the history of chronic glomerulonephritis and the patient's refusal. She was maintained on HD since that time. She was transferred to our center at the 20^th ^week of gestation where HD was performed daily for 6 hours/session.

An eKt/V of 2 was achieved (the patient was passing 350 ml urine/day with estimated GFR of 4.07 ml/min/1.73 m^2^) with a mean pre-dialysis BUN of 12.65 mg/dl. Her nPCR was 1.24 and blood gas analysis revealed a pH of 7.35, HCO3 of 23.1 meq/L, serum calcium 2.23 mmol/L and serum phosphate 1.52 mmol/L. Her medications consisted of; ά methyl dopa (500 mg TID – with dose adjustment when necessary for tight BP control: below 120/80 mmHg), folic acid 5 mg OD and calcium carbonate (600 mg TID). As expected, erythropoietin and iron requirements were increased during her pregnancy (erythropoietin from a weekly dose of 6000 to a mean of 14000 units and iron saccharate to 100 mg IV once every week instead of 100 mg/day of oral ferrous fumarate). Her serum albumin ranged between 2.8 and 3 gm/dl and her hemoglobin ranged between 8.33 and 9.74 g/dl.

Following discussion with the obstetric team, in both cases programmed adjustment of the dry weight was done by revising the estimated dry weight weekly to an expected weight gain during progression of pregnancy. In addition to routine pregnancy care, fetal well-being was monitored by way of serial ultrasound assessment of biophysical profiles, Doppler studies and estimated fetal weights. At 32^nd ^week's gestation a diagnosis of pre-eclampsia (based on uncontrolled blood pressure – despite increased dose of ά-methyldopa – and development of proteinuria) was settled. The patient was electively delivered by cesarean section resulting in a single viable girl – weighing 1.7 kg – with an uneventful neonatal period.

## Discussion

In 1971 Confortini et al. [1] reported the first successful pregnancy in a woman on chronic HD.

Recent publications report pregnancy in 1–7% in women on chronic dialysis [2]. Moreover, pregnancy in contemporary women on dialysis is more likely to be successful, with 30–50% of pregnancies resulting in delivery of a surviving infant [3].

The results of a survey of pregnancy in the HD population of the Kingdom of Saudi Arabia (over 5 years – 1985 to 1990) showed a frequency of 7% (27 among 380 women on HD) with 37% successful outcome (10 patients) [4].

Early diagnosis of pregnancy in ESRD requires careful attention. Irregular menstrual cycles, amenorrhea, nausea and elevated beta-subunit of human chorionic gonadotropin have been observed in some patients with renal failure which may give a false-positive pregnancy test. A late diagnosis delays the intensive antenatal care and reduces the successful outcome [5].

In one of our cases, the symptoms of pregnancy were first attributed to inefficient dialysis before pregnancy was diagnosed. As urine testing for pregnancy is not reliable in patients with chronic renal failure because of altered renal clearance and the difference in the molecular forms of beta-subunit of human chorionic gonadotropin measured by different assays [6]. As recommended, we used abdominal sonography to confirm pregnancy and assess gestational age as soon as we were informed about the pregnancy. Therefore, in such cases we suggest a blood pregnancy test [to estimate B subunit of human chorionic gonadotrophin (HCG) in blood] to be done prior to any abdominal x-ray if there an abdominal complaint.

The number of successful pregnancies in dialysis patients has improved over the years [7]. The outcome is better in patients who conceived before starting dialysis compared with those who became pregnant while on dialysis [3].

In our view, these figures should be interpreted with caution for a number of reasons. Firstly, there are no comprehensive prospective studies of conception among women with ESRD. Secondly, the literature addressing pregnancy in women on dialysis is composed primarily of survey studies, single center retrospective reviews, and case reports. Thirdly, pregnancies ending in the first or second trimester by elective or spontaneous abortions are variably included, thus reporting bias may confound the results.

Since the 1980s, the infant survival rate has improved from 20–30% [8] up to 50% in 2003 [2]. This is probably due to the care provided by a multidisciplinary management team, characterized by close collaboration between patients, nephrologists, dialysis staff, obstetricians and neonatologists.

Despite improved infant survival, half of pregnancies in women on dialysis are not successful and the proportion of neonatal deaths remains higher than in the general population. Infants born to women on dialysis are usually premature, with an average gestational age of 32 weeks. Our finding is in agreement with earlier reports regarding gestational age since we failed to prolong gestational age beyond 32 weeks, despite the maximum multidisciplinary care we tried to provide.

Multiple causes of premature delivery exist, including polyhydramnios, maternal hypertension and premature rupture of the membranes [9]. Since increasing dialysis frequency lowers predialysis BUN levels, adequate dialysis may reduce the occurrence of polyhydramnios and thus lower the risk of premature labor [5]. Increasing the dialysis dose prolongs gestation, resulting in a higher infant birth weight and thus an infant with better chance of survival [4].

Despite the fact that no randomized prospective trials of pregnant women on dialysis exist, retrospective data suggest maintaining predialysis BUN values – beyond 16 to 20 weeks – at ≤ 50 mg/dl is an appropriate goal [5]. Pregnant women on dialysis will generally require 16–24 hours of HD each week.

In one series, fetal mortality was directly proportional to maternal BUN level, with no successful pregnancies occurring in patients with BUN levels greater than 60 mg/dL [2]. In our cases the mean pre-dialysis BUN was maintained at 15.13 mg/dl and 12.65 mg/dl respectively during pregnancy, which may have contributed in part to the successful outcome.

In the largest study to date, the Registry for Pregnancy in Dialysis Patients reported a significant correlation between hours spent on dialysis therapy and improved fetal outcome. The increase in dialysis time seems to improve the pregnancy outcome and offer several advantages: It ensures less uremic environment to the fetus and allows the mother more liberal diet (Potassium and protein), it may help to control hypertension and fluid intake and may also reduce the amplitude of blood voulme and electrolyte shifts [3]. This is consistent with our results as in both cases dialysis treatment was intensified (up to daily dialysis in one case) resulting in viable mature babies.

Estimating appropriate target weights for pregnant women on dialysis may be difficult. Allowances must be made for fetal and placental growth as well as the 30% increase in plasma volume that occurs with pregnancy. After the first trimester, weight gain is usually linear and is approximately 1 pound/week. Ultrafiltration goals can be adjusted based on this expected pregnancy-induced weight gain [9].

Similarly dialysate adjustment may be needed to maintain appropriate levels of serum calcium and to avoid hypocalcemia and/or post-treatment hypercalcemia. Since the placenta converts some 25-hydroxyvitamin D3 to 1, 25-dihydroxyvitamin D3, adjustment of vitamin D may be required during pregnancy and should be guided by measurement of levels of vitamin D, parathyroid hormone, calcium and phosphorus [10].

Anemia occurs during pregnancy and pregnant dialysis patients require intensive anemia management. Erythropoietin has been given safely to pregnant dialysis patients [10]. Erythropoietin doses need to be increased by approximately 50% in order to maintain target hemoglobin levels of 10–11 g/dl. The reason for the higher erythropoietin doses is unknown, but increased vascular volume with subsequent hemodilution and possibly erythropoietin resistance (due to enhanced cytokine production) during pregnancy may contribute [10]. This is consistent with our observation, as erythropoietin doses were increased (by more than 70% and 100% in case 1 and 2 respectively) to maintain hemoglobin level comparable to that before pregnancy.

In addition, both intravenous iron [5] and heparin appear to be safe during pregnancy however frequent monitoring of iron stores is required and minimizing heparin dose is recommended [10].

Hypertension is the most frequently reported maternal complication in this population, occurring in 42–80% of these women [11]. Antihypertensive medications are often required to maintain maternal diastolic blood pressure in the 80–90 mmHg range [9]. The mainstays of treatment are methyldopa, B-blockers, and hydralazine. In cases of severe hypertension, clonidine and calcium channel blockers have been used safely [11]. In one of our cases, hypertension was difficult to control after 30 weeks of gestation despite maximum dose of methyldopa, necessitating elective termination.

However, the other case remained normotensive without any antihypertensive medications throughout pregnancy with intensified dialysis.

There is little information on the nutritional status of pregnant dialysis patients; however 1 g/kg/day protein intake plus an additional 20 g/day for fetal development have been suggested [11]. Folate supplementation is required, particularly early in fetal development and replacement of water-soluble vitamins should be continued during pregnancy [11].

Maternal mortality is very low and rarely reported [3,4]. Cesarean section delivery is common among women on dialysis and is most often prompted by premature rupture of membranes.

In conclusion, we hereby report two cases of successful pregnancy in 2 Saudi patients, the first case with chronic renal failure maintained on chronic hemodialysis and the second with pre-existing renal disease aggravated by pregnancy. We advise that all aspects of dialysis, including duration, adequacy, nutrition, anemia, calcium and phosphate metabolism and BP control needs to be closely followed throughout the course of pregnancy. Furthermore, a successful pregnancy in woman on dialysis requires collaboration among nephrologists, dialysis unit staff and obstetricians. Finally, since pregnancy can occur in woman on dialysis, health care providers should discuss fertility and contraception with their premenopausal dialysis patients.

## Abbreviations

ESRD: End Stage Renal Disease.

HD: Hemodialysis.

PSCKD: Prince Salman Center for kidney disease.

## Competing interests

The author(s) declare that they have no competing interests.

## Authors' contributions

KA-S: has been involved in drafting the manuscript and revising it critically for important intellectual content.

AS: have made substantial contributions to conception and design or acquisition of data, analysis and interpretation.

All authors given final approval of the version to be published.

## Consent

Written informed consent was obtained from the patients before publication of this case series. A copy of the consent is available for review by the Editor-in-Chief of this journal.
